# Restoring leptin sensitivity in metabolic and extra-metabolic leptin resistance: Pharmacologic strategies, systemic implications, and future directions

**DOI:** 10.17179/excli2026-9420

**Published:** 2026-05-15

**Authors:** Milan Obradovic, Sara Khodahemmati, Sonja Zafirovic, Esma R. Isenovic

**Affiliations:** 1 Department of Radiobiology and Molecular Genetics, Vin?a Institute of Nuclear Sciences, National Institute of the Republic of Serbia, University of Belgrade, Belgrade, Serbia

**Keywords:** leptin, leptin sensitivity, leptin resistance, leptin therapy

## Abstract

The presence of leptin resistance is a key barrier to the successful management of obesity. The most recent study has focused on pharmacological drugs that are associated with inflammation and endoplasmic reticulum stress, as well as inhibitors of negative regulators, including PTP1B, SOCS3, and HDAC6, and substances that affect the mTOR and cAMP/Epac/Rap1 signaling pathways. Having an understanding that leptin resistance is caused by defective leptin signaling pathways, such as a disruption in the JAK-STAT pathway or an increase in the expression of SOCS3, shows the difficulty of restoring sensitivity. The physiological implications of leptin resistance extend beyond the regulation of metabolism and can influence the cardiovascular system, the liver (NAFLD/MASLD), the immunological system, the inflammatory system, reproductive health and the neuroendocrine system. The fact that leptin has metabolic impacts on various systems highlights the importance of taking an integrated strategy to treatment procedures. Within the context of metabolic and extra-metabolic leptin resistance, this review provides a summary of recent literature evidence pertaining to the restoration of leptin responsiveness. Also, we include a discussion regarding pharmacologic strategies, systemic implications, and future directions.

See also the graphical abstract(Fig. 1).

## 1. Introduction

Most people with obesity exhibit alteration of circulating levels of leptin, and poorly respond to its appetite-suppressing and weight-reducing effects. Beyond its primary functions, leptin acts as a molecular connector between metabolic status and immune regulation as well as a mediator of vascular inflammation (Zhao et al., 2021[72]). Early-life severe obesity is predisposed to individuals with leptin deficiency or defective leptin receptors. Studies have also linked leptin malfunction to cardiometabolic diseases, including type 2 diabetes, cardiovascular disease, liver steatosis, as well as to the dysregulation of the immune system (Poetsch et al., 2020[45]; Besci et al., 2023[3]). This issue can be dramatically reversed by leptin replacement, which explains the central effect of leptin on maintaining energy homeostasis (Farooqi and O'Rahilly, 2014[19]). Evidence indicates that leptin resistance could be the culprit for the poor effects of leptin therapy in obese individuals. Recent research has identified new cellular and molecular agents that modulate leptin resistance and sensitization (Genchi et al., 2021[22]). Also, it has expanded knowledge of leptin's functions beyond just controlling appetite. Mechanistic studies now suggest that increased mammalian target of rapamycin (mTOR) activity in proopiomelanocortin (POMC) neurons is necessary for the weight-reducing effects of leptin. Using rapamycin or genetically modifying mTOR components can restore leptin responsiveness and reduce fat in experimental animal models (Cota et al., 2006[9]). There are observations suggesting that cAMP-dependent Epac-Rap1 signaling and gut-derived glucose-dependent insulinotropic polypeptide (GIP)/Rap1 activation in the hypothalamus promote suppressor of cytokine signaling 3 (SOCS3) induction and impair POMC neuron responses, inducing central leptin resistance (Hu et al., 2025[26]). In addition, both human and animal studies suggest peripheral leptin resistance in the liver, adipose tissue, and skeletal muscle, which is associated with insulin resistance (Cruz-Color et al., 2020[10]; De la Cruz-Color et al., 2024[12]).

The translational studies have been looking at molecular mechanisms that may trigger leptin resistance to make the body respond better to leptin (Tan et al., 2025[59]). In this review, we extensively assessed the therapeutic strategies ranging from leptin replacement in leptin-deficient states to leptin sensitizers and rational combination regimens in common obesity. Within the context of metabolic and extra-metabolic leptin resistance, this review provides a summary of recent literature evidence pertaining to the restoration of leptin responsiveness. Also, we included a discussion regarding pharmacologic strategies, systemic implications, and future directions.

## 2. Cardiometabolic and Extra-Metabolic Implications of Leptin Resistance

Leptin functions as a primary conductor of glucose and lipid homeostasis by modulating insulin sensitivity, mitochondrial function, and energy partitioning across tissues. Besides, it suppresses gluconeogenesis and promotes fatty acid oxidation (Roy et al., 2025[50]). Thus, in the healthy state, leptin promotes metabolic flexibility. In states of obesity, this synchronized system becomes disrupted and effectively mutes the beneficial effects of leptin. Consequently, hyperleptinemia as well as leptin resistance contribute to widespread disruption of nutrient metabolism and decreased sensitivity of insulin (Roy et al., 2025[50]). Leptin resistance changes leptin's nature from a largely homeostatic hormone into a factor of cardiometabolic and systemic dysfunction (Roy et al., 2025[50]). Thus, impaired cellular responses to leptin are linked with the development of cardiovascular disease, fatty liver disease, chronic inflammation, and reproductive axis dysregulation (Figure 2(Fig. 2)).

Leptin resistance led to multisystem signaling failure, which may help explain why obesity increases morbidity and mortality even with only modest weight change (Hernández-Díaz et al., 2024[25]). On the contrary, restored leptin sensitivity showed benefits in various physiological systems, which sets leptin resistance as a central mediator of multisystem pathology in obesity (Mendoza-Herrera et al., 2021[38]). This provides a promising therapeutic strategy for obesity-related diseases rather than weight loss alone.

### 2.1 Cardiovascular system

Leptin exerts multiple effects in the cardiovascular system, including vascular homeostasis through stimulation of nitric oxide production and promotion of endothelial function (Polyakova et al., 2021[46]). In pathological conditions such as obesity, the beneficial effects of leptin are reduced, while pro-sympathetic, pro-hypertensive, and pro-atherogenic signaling pathways are increased (Gutiérrez-Cuevas et al., 2021[24]; Polyakova et al., 2021[46]). Thus, leptin transforms from a homeostatic factor into a catalyst of sympathetic overactivity and vascular dysregulation.

Furthermore, chronic hyperleptinemia promotes the progression of hypertension, left ventricular hypertrophy, and heart failure (Gutiérrez-Cuevas et al., 2021[24]; Polyakova et al., 2021[46]). Although leptin's capacity to stimulate renal sympathetic nerves remains intact, sodium retention increases blood pressure (Laule and Rahmouni, 2025[31]; Theodorakis and Nikolaou, 2025[61]). In addition, hyperleptinemia is correlated with adverse cardiac remodeling, increased carotid intima-media thickness, and a higher incidence of cardiovascular events in individuals with obesity and type 2 diabetes (Vilariño-García et al., 2024[64]).

Leptin has diverse effects on atherogenesis. It accelerates atherosclerosis by acting on endothelial cells, vascular smooth muscle cells, and macrophages, where hyperleptinemia promotes oxidative stress, endothelial dysfunction, vascular inflammation, smooth muscle cell proliferation, and arterial stiffness (Raman and Khanal, 2021[47]; Vilariño-García et al., 2024[64]). Pro-inflammatory activity of leptin in macrophages promotes foam cell formation (Pereira et al., 2021[44]), which increases the risk of plaque rupture (Vilariño-García et al., 2024[64]). In addition, increased leptin levels are associated with adverse cardiovascular outcomes in coronary artery disease. These findings support that leptin resistance serves as an independent cardiometabolic risk (Raman and Khanal, 2021[47]). Besides affecting blood vessel structure, leptin also influences thrombosis and the stability of plaques in the arteries. It was shown that leptin augments platelet aggregation, increases tissue factor expression, and amplifies inflammatory signaling within the vascular wall (Polyakova et al., 2021[46]; Vilariño-García et al., 2024[64]; Zawadzka et al., 2025[70]). These mechanisms create a pro-coagulant state that predisposes the vasculature to rupture and acute thrombotic events (Zawadzka et al., 2025[69]).

### 2.2 Liver and NAFLD/MASLD

Hepatic leptin resistance is widely acknowledged as a major risk factor of the MASLD spectrum (De la Cruz-Color et al., 2024[12]). It encourages the development of more advanced inflammation and fibrosis, indicating that intact leptin signaling in hepatocytes is essential. Also, it controls lipogenesis and helps maintain lipid homeostasis (Huang et al., 2022[27]). Disruption of hepatic leptin signaling impairs fatty acid oxidation, promotes triglyceride accumulation, and exacerbates insulin resistance (De la Cruz-Color et al., 2024[12]). Clinical and biopsy-based studies show that patients with steatohepatitis have increased circulating leptin and upregulated hepatic SOCS3 (De la Cruz-Color et al., 2024[12]). The amount of SOCS3 in the liver is linked to lipogenic genes such as SREBF1 and SCD1 (De la Cruz-Color et al., 2024[12]). This pattern indicates a detrimental condition in which leptin's normal metabolic functions are reduced. At the same time, SOCS3-dependent fat production and inflammatory signaling persist (De la Cruz-Color et al., 2024[12]).

Leptin affects hepatic stellate cell activation and fibrogenesis (Saxena et al., 2002[52]). However, decreased leptin sensitivity fails to regulate lipid metabolism, while its capacity to stimulate hepatic stellate cells for collagen production remains intact (Saxena et al., 2002[52]). Thus, leptin resistance creates a harmful signaling asymmetry of leptin. In addition, the liver is unsensitive to leptin's metabolic benefits but remains sensitive to its fibrogenic inputs (Martínez-Uña et al., 2020[37]). This functional disconnect shows how chronic hyperleptinemia in obesity subverts the liver's healing response.

Peripheral leptin resistance alters lipid storage and release in adipose tissue, promoting accumulation of fat in non-adipose organs such as the liver (De la Cruz-Color et al., 2024[12]). Thus, leptin resistance further exacerbates systemic metabolic dysfunction by disrupting mitochondrial function and nutrient metabolism, thereby intensifying insulin resistance (Wang et al., 2025[66]). Together, these defects show the systemic nature of leptin resistance and are an important factor in promoting liver disease.

### 2.3 Immune and inflammatory pathways

Leptin functions as an important immunomodulatory cytokine, which stimulates innate and adaptive immune responses (Abbasi Kasbi et al., 2026[1]). Leptin affects T-cell proliferation and activity and promotes macrophage activation and cytokine production. This functional coordination means that immune activity mirrors energy availability (Kiernan and MacIver, 2020[28]). However, obesity subverts this regulatory coordination. The resulting chronic hyperleptinemia amplifies the inflammatory signature of macrophages and T cells and promotes systemic inflammation and tissue stress (Abbasi Kasbi et al., 2026[1]). In addition, leptin resistance disrupts immune regulation in a tissue-specific manner. Metabolic tissues do not respond to leptin's homeostatic signals, while pro-inflammatory effects remain activated. The production of inflammatory mediators, such as TNF-α and IL-6, as well as other cytokines, is stimulated by this selective response (Kiernan and MacIver, 2020[28]). Increased leptin levels in adipose tissue stimulate macrophage activation and shift macrophage polarization toward pro-inflammatory M1-like phenotypes (Abbasi Kasbi et al., 2026[1]). This adipose-immune interaction worsens systemic inflammation and cardiometabolic pathology (Saitoh et al., 2021[51]; Roy et al., 2025[50]). In addition, hyperleptinemia is associated with hypothalamic inflammation that effectively mutes leptin signaling at its source. This creates a cycle in which immune activation and metabolic dysfunction perpetuate each other (Engin 2024[16]). Together, these interactions position leptin resistance as both a cause and a consequence of obesity related inflammation (Engin 2024[16]). They merge immune dysregulation into the pathophysiology of metabolic and cardiovascular disease (Roy et al., 2025[50]; Tilg et al., 2025[62]).

### 2.4 Reproductive and neuroendocrine consequences of leptin resistance

Leptin is a necessary permissive signal for normal reproductive function via the hypothalamic-pituitary-gonadal (HPG) axis (Stefanakis et al., 2024[55]), and its absence in congenital leptin deficiency, severe hypogonadotropic hypogonadism occurs, characterized by delayed puberty and infertility. This condition is readily reversed by leptin replacement (Stefanakis et al., 2024[55]). Observations suggest that leptin actively regulates reproduction rather than simply reflecting body fat (Childs et al., 2021[6]). Compared with leptin deficiency, obesity is characterized by increased leptin levels and leptin resistance that disrupts reproductive signaling even with high circulating leptin (Childs et al., 2021[6]; Hu et al., 2025[26]). Leptin resistance has been linked to impaired reproductive function in both sexes (Childs et al., 2021[6]). Altered leptin signaling interferes with gonadotropin-releasing hormone (GnRH) secretion, which further disrupts the regulation of luteinizing and follicle-stimulating hormone (Obaideen et al., 2024[40]). In obese men, increased leptin levels are often related to lower testosterone and poorer semen parameters (Obaideen et al., 2024[40]). In obese women, leptin resistance is most often associated with polycystic ovary syndrome (PCOS). However, this may be attributed to coexistence with insulin resistance rather than leptin resistance itself (Reesor et al., 2024[48]).

## 3. Pharmacologic Strategies to Restore Leptin Sensitivity

Pharmacological leptin sensitizers seek to restore the normal signaling pathways even when leptin levels are already high. This would allow endogenous or exogenous leptin to reactivate anorexigenic and metabolic pathways in diet-induced obesity (Pena-Leon et al., 2024[42]). There is limited information available from human studies (Engin 2024[16]). The existing report is primarily from early-phase trials (Tam et al., 2011[58]; ERX Pharmaceuticals, 2023[17]; Pena-Leon et al., 2024[42]).

**Figure 1:**
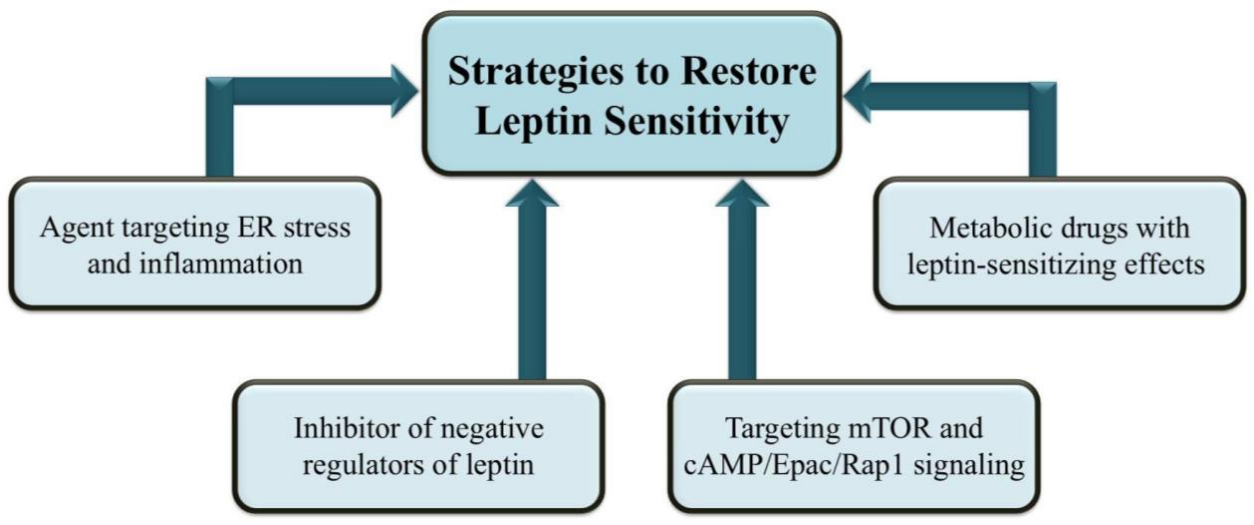
Graphical abstract: Strategies to restore leptin sensitivity. Arrows from each box point to the central concept, representing convergent mechanisms through which these strategies restore leptin signaling. Agents targeting ER stress and inflammation reduce hypothalamic ER stress and pro-inflammatory kinase activity (e.g., JNK, IKKβ), thereby improving leptin receptor function. Inhibitors of negative regulators block endogenous brakes on leptin signaling, including SOCS3, PTP1B, and HDAC6, to sustain JAK2-STAT3 pathway activation. Modulation of mTOR and cAMP/Epac/Rap1 signaling tunes intracellular nutrient-sensing and second-messenger pathways that gate leptin responsiveness in hypothalamic neurons. Metabolic drugs with leptin-sensitizing effects, such as GLP-1 receptor agonists and amylin analogs, enhance central leptin action through mechanisms that may be partly independent of weight loss. ER, endoplasmic reticulum; SOCS3, suppressor of cytokine signaling-3; PTP1B, protein tyrosine phosphatase 1B; HDAC6, histone deacetylase 6

**Figure 2:**
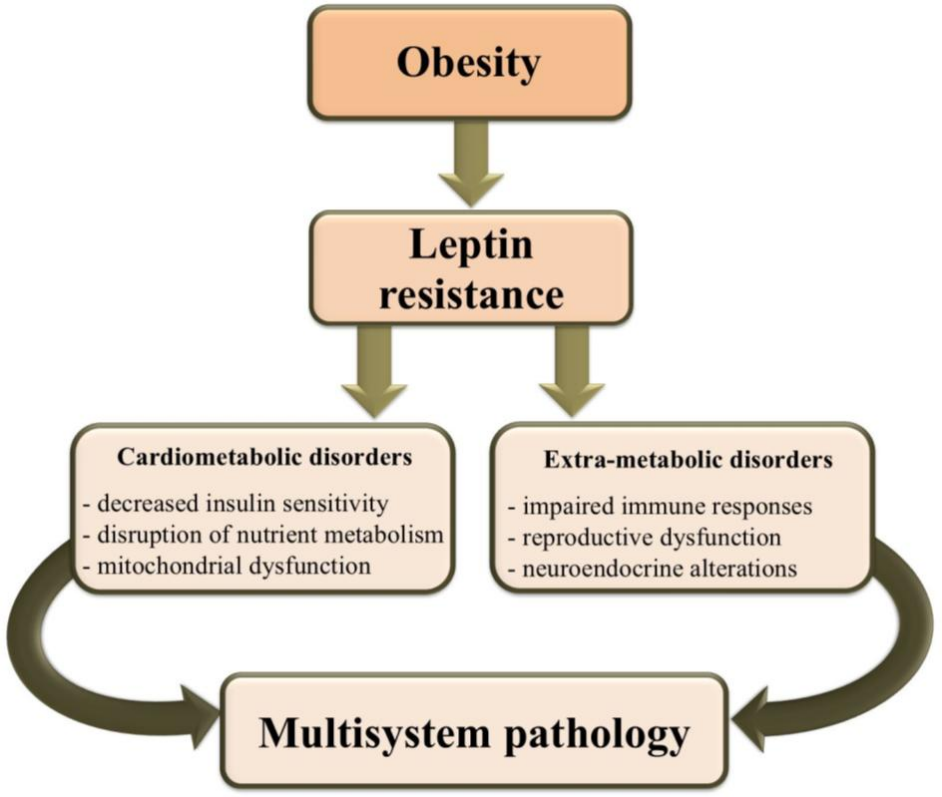
Pathology of leptin resistance in obesity. Hyperleptinemia and decreased leptin sensitivity induce cardiometabolic and extra-metabolic disorders, which eventualy progress into multisystem pathology.

Pharmacologic strategies to restore leptin sensitivity are presented in Figure 1(Fig. 1). This field is still largely in the research phase rather than being a standard clinical practice.

### 3.1 Agents targeting ER stress and inflammation

By reducing hypothalamus ER stress and low-grade inflammation, several small compounds can increase leptin sensitivity (Dong et al., 2025[14]; Marinho et al., 2026[36]). Comprehensive screening was used to identify the triterpenoid celastrol. It was shown in obese models to be an effective leptin sensitizer that restores leptin responsiveness (Liu et al., 2024[35]). Besides celastrol, withaferin A, the steroidal lactone, can promote leptin‑induced STAT3 phosphorylation and glycemic control, functioning as a leptin sensitizer (Lee et al., 2016[32]; Ya et al., 2024[68]). Both celastrol and withaferin A can alleviate decreased leptin signaling by inhibiting inflammatory kinases and altering chaperone pathways (Lee et al., 2016[32]). The pleiotropic nature and toxicity profile of these molecules demand the development of more refined DDS (Sun et al., 2024[56]). Alternative strategies focus on deactivating the inflammatory kinases, such as c-Jun N-terminal kinase and IκB kinase, which are activated by overeating and promote inflammation and interfere with leptin and insulin signaling (Roy et al., 2025[50]). These kinases decreased the leptin signal through SOCS3 and promoted the inhibitory serine phosphorylation of main signaling components (Suren Garg et al., 2023[57]; Fernández-González et al., 2026[20]). Furthermore, inhibition of stress-activated pathways has been shown to quell hypothalamic turbulence, normalize the JAK2-STAT3 and PI3K axes and decrease weight gain (Roy et al., 2025[50]). Similarly, chemical chaperones such as tauroursodeoxycholic acid also restore leptin sensitivity and body weight control, although solid human clinical observations in obesity are still lacking (Roy et al., 2025[50]).

### 3.2 Inhibitors of negative regulators of leptin signaling

As SOCS3 and protein tyrosine phosphatase 1B (PTP1B) are central negative regulators of leptin signaling (Hu et al., 2025[26]). An extensive effort has been devoted to clarifying the mechanism of silencing these molecules. Leptin-induced STAT3 activation is amplified, diet-induced obesity is prevented, and glucose homeostasis is restored when PTP1B is genetically deleted in LepRb neurons (Tsou et al., 2012[63]). It was shown in experimental studies that inhibitors of PTP1B decrease weight gain and restore leptin and insulin signaling; however, their clinical application is limited by their lack of selectivity and effects on other pathways (Delibegović et al., 2024[13]). Given the limitations of direct SOCS3 inhibition, the focus has been directed to indirect modulation of its action (Münzberg et al., 2024[39]). One strategy includes inhibition of histone deacetylase 6 (HDAC6) (Hu et al., 2025[26]). In obese mice, selective HDAC6 inhibitors increased the amount of leptin receptors on the cell surface, strengthened leptin signaling in the hypothalamus, and produced extra weight loss when combined with leptin (Çakır et al., 2022[4]; Guan et al., 2024[23]). These observations suggest that epigenetic or post-translational modulation of negative regulators could serve as a viable pharmacologic strategy to restore leptin sensitivity, pending toxicity and safety evaluation.

### 3.3 Modulation of mTOR and cAMP-Epac-Rap1 pathways

Overactivation of mTOR in POMC neurons contributes to leptin resistance. Thus, targeting mTOR signaling emerged as a primary therapeutic target to improve leptin function (Hu et al., 2025[26]; Tan et al., 2025[59]). In diet-induced obese models, rapamycin and its analogs successfully re-established the JAK2-STAT3 signaling that reduced hyperphagia and reversed adipose accumulation (Cota et al., 2008[8]; Tan et al., 2025[59]). These observations highlight mTOR as a drug target for leptin sensitization (Tan et al., 2025[59]). Considering that systemic mTOR inhibition is limited by immunosuppressive effects, dyslipidemia, and glucose intolerance in humans, this indicates the basis for a brain-directed or neuron-selective strategy to clinical application (Yang et al., 2012[69]; Konopka et al., 2023[30]; Tan et al., 2025[59]). Other pathways, such as the cAMP-Epac-Rap1 axis, were also being examined to restore leptin signaling (Chen et al., 2026[5]). Research in rodent models suggests that silencing the SOCS3 and PTP1B pathways can prevent weight gain and restore leptin signaling. Accordingly, pharmacologic modulation of the Epac-Rap1 axis with small-molecule modulators is a promising translational strategy to unmask the anorectic capacity of leptin (Chen et al., 2026[5]). However, such compounds are still at an early research stage and have not yet been evaluated for obesity treatment.

### 3.4 Metabolic drugs with leptin-sensitizing effects

Some established metabolic drugs display leptin-sensitizing properties in preclinical models (Kim et al., 2006[29]; Lee et al., 2016[32]; Liu et al., 2024[35]). Metformin has been reported to restore leptin sensitivity in high-fat-fed obese rats (Tang et al., 2016[60]; Hu et al., 2025[26]). It is improving leptin-induced anorexia and hypothalamic STAT3 activation while reducing weight gain and insulin resistance. These effects appear to involve AMPK activation, reduced hypothalamic inflammation, and restored insulin signaling (Kim et al., 2006[29]). This illustrates that common antidiabetic agents may exert secondary leptin-sensitizing actions. Similarly, GLP-1 receptor agonists and dual agonists (GLP 1/GIP) reduce hypothalamic inflammation and ER stress (Dong et al., 2025[14]; Marinho et al., 2026[36]). They may indirectly increase leptin responsiveness, which is one rationale for testing them in combination with leptin or leptin analogues (Dong et al., 2025[14]).

### 3.5 Translational status and challenges

A common observation across these pharmacologic strategies is the benefit of combining leptin sensitizers with leptin in preclinical models. This often achieves greater weight loss and metabolic restoration than using either strategy alone (Obradovic et al., 2021[41]; Pena-Leon et al., 2024[42]; Fu et al., 2025[21]). However, translating these observations to clinical application is difficult. Numerous sensitizers, like celastrol and withaferin A, have narrow safety margins and can affect other pathways in the body (Liu et al., 2024[35]; Ya et al., 2024[68]). Also, central regulators such as mTOR and Epac-Rap1 are widespread for multiple physiological processes (Chen et al., 2026[5]). Also, we lack reliable markers to measure leptin sensitivity in clinical trials. Emerging development will demand targeted delivery of drugs specifically to the brain, using precisely selective inhibitors, and carefully selecting patients based on factors like their genetic background or baseline leptin sensitivity. This will be necessary to use these mechanistic insights in the treatment of obesity safely.

## 4. Gaps, Challenges, and Future Directions

While major advances have been made in understanding leptin biology, remaining gaps in leptin signaling in pathological states limit the clinical success of leptin therapies in obesity and related metabolic diseases, as its regulation, reversibility, and individual differences remain poorly defined. Responding to these limitations will advance the therapeutic potential of leptin-related drugs.

### 4.1 Lack of reliable biomarkers of leptin sensitivity

A major barrier to clinical translation is the lack of validated biomarkers for measuring leptin sensitivity in humans. Circulating leptin concentrations closely reflect adiposity, but show little information about leptin responsiveness at the cellular or tissue levels, making it difficult to find individuals who may benefit from leptin-based or leptin-sensitizing therapies (Chiriacò et al., 2023[7]). Therefore, there is still a diagnostic barrier that affects current attempts to measure leptin sensitivity. Other markers, like soluble receptor levels, as well as the leptin to adiponectin ratio, provide only indirect information instead of underlying biological signaling pathways (Engin 2024[15]). So far, experimental techniques, including neuroimaging of hypothalamic responses, ex vivo leptin-stimulated STAT3 phosphorylation assays, and SOCS3 or PTP1B expression in relevant tissues, give a mechanistic understanding. However, unfortunately, they are not yet standardized for routine clinical use (Liu et al., 2021[34]; Roger et al., 2022[49]; Perakakis and Mantzoros, 2024[43]). In order to classify patients according to leptin sensitivity and follow-up responses in sensitizer trials, new attempts should focus on finding biomarker panels that could include circulating markers, genetic data, and imaging results.

### 4.2 Translational limitations of preclinical models

Much of the current mechanistic recognition of leptin resistance comes from rodent models of diet-induced obesity. While these models have been helpful for recognition of the molecular pathways involved, they have limitations when it comes to translating these observations to humans. These limitations are due to species differences in hormonal regulation, neuroanatomy, and metabolism. Although multiple leptin-sensitizing compounds show beneficial short-term efficacy in animals (Kim et al., 2006[29]; Lee et al., 2016[32]; Liu et al., 2024[35]), their clinical use requires long-term safety tests. In addition, targeting mTOR, Epac-Rap1, and inflammatory kinases raises worries given their involvement in various processes in the organism (Schmidt et al., 2013[53]; Lezoualc'h et al., 2016[33]; Zhang et al., 2023[71]). More sophisticated translational systems are needed to uncover these limitations, such as organoids, human neuronal models, and early-phase clinical trials that are designed around mechanistic endpoints rather than weight loss alone.

### 4.3 Safety and specificity of central leptin pathway modulation

Modulating leptin signaling in the brain has safety risks because central leptin signaling involves opposing physiological consequences. Besides modulating metabolic function, restoring leptin sensitivity could affect immune function, growth, and cellular regulation (de Candia et al., 2021[11]). It was shown in an animal model that systemic mTOR inhibition reverses leptin resistance in hypothalamic POMC neurons (Tan et al., 2025[59]); however, this may not be suitable in humans. Consequently, future strategies should focus on tissue specificity and modification of selective leptin pathways. Besides, more selective modulation of leptin signaling may be possible by the development of gene‑based strategies, nanoparticle‑based BBB penetration, as well as improvement of CNS‑selective drug delivery systems (Wang et al., 2025[67]).

### 4.4 Inter-individual heterogeneity and precision medicine

Leptin resistance is different between individuals with various pathological manifestations, including impaired BBB transport, hypothalamic inflammation, ER stress, SOCS3 or PTP1B overactivity, mTOR dysregulation, or peripheral tissues resistance (Obaideen et al., 2024[40]). Furthermore, genetic variability in the LEP and LEPR, along with variations in downstream JAK-STAT and melanocortin circuitry, dictates an individual's differences (Fairbrother et al., 2018[18]; Šket et al., 2022[54]). Thus, improved diagnostics to help identify these differences are necessary for shifting away from generalized to personalized strategies matching specific biological signatures of the patient. Most clinical trials do not stratify participants based on mechanistic or genetic profiles, thereby concealing meaningful benefits in responsive subgroups. By failing to stratify participants based on their distinct mechanistic or genetic signatures, treatments may neglect these prime benefits. The next steps demand a transition to a precision medicine structure with specific leptin-responsive interventions.

### 4.5 Translational gap for leptin sensitizers

Although a range of leptin sensitizers, such as withaferin A, celastrol, and inhibitors of the PTP1B and mTOR axes, are efficient in experimental models, they cannot currently be applied to humans (Lee et al., 2016[32]; Liu et al., 2024[35]). Restoring leptin signaling was documented in rodents, but it still cannot be transferred into late-phase human trials (Engin, 2024[15]). Numerous obstacles, including limited treatment windows, systemic toxicity, and the practical challenge of attaining selective brain penetration, contribute to this stagnation. Even though the long-term safety of modulating such ubiquitous signaling components remains a major clinical barrier, this demands a more cautious and refined strategy for drug development. Translation of leptin research into clinical practice will depend on a phased development strategy. Currently, widely used metabolic drugs with leptin-sensitizing properties, such as metformin or GLP-1/GIP agonists, are an approach worthy of further research (Hu et al., 2025[26]).

### 4.6 Rethinking therapeutic targets: Partial leptin reduction and downstream pathways

The partial reduction of leptin signaling in hyperleptinemic and obesity states may restore leptin sensitivity (Zhao et al., 2019[73]). This approach of metabolic recalibration of leptin is supported by experimental findings showing that decreasing leptin to its physiological levels may reduce the inhibitory influence of SOCS3 and PTP1B (Zhao et al., 2019[73]). By clearing the molecular noise of chronic overstimulation, it may be possible to reset the leptin axis and re-establish signaling sensitivity. However, strategies for safely achieving and sustaining partial leptin reduction in humans demand prospective investigation.

In parallel, the clinical success of melanocortin-4 receptor (MC4R) agonists such as setmelanotide in rare genetic leptin melanocortin disorders shows the benefit of modulating downstream pathways rather than leptin itself (Hu et al., 2025[26]). New therapies may combine mild central leptin sensitization with activation of leptin in peripheral tissues to avoid adverse sympathetic or inflammatory effects.

### 4.7 Integration with cardiometabolic and extra-metabolic risk

The transition toward a systemic model of obesity demands movement of leptin resistance from the periphery of research to the center of the patient treatment approach. Although leptin resistance is tightly associated with numerous disorders, it remains a major problem for cardiovascular and liver guidelines (Wang et al., 2023[65]; Adamowski et al., 2024[2]; Wang et al., 2025[66]). Thus, extensive epidemiological studies should be conducted to test whether improving leptin resistance can prevent cardiovascular events and liver-related morbidity.

## 5. Conclusions

Growing evidence indicates that improving leptin sensitivity should be transformed from monotherapy to a drug combination. Current translational efforts focus on decreasing cellular burdens of ER stress and inflammation that occur in obese people with leptin resistance. Whether through the inhibition of intracellular inhibitors or the application of synergistic incretin-based strategies, the goal has shifted toward restoring signaling integrity. In emerging leptin-based therapy, the priorities are to develop clinically feasible biomarkers of leptin sensitivity, design safe and selective leptin-sensitizing agents, and the stratification of patients based on mechanistic and genetic features to match the right treatment with the right individual. Further therapeutic strategies should provide broader access that involves a combination of leptin sensitizers, metabolic drugs, and modulators of downstream pathways such as MC4R agonists.

Thus, increasing leptin sensitivity becomes a goal affecting beyond body weight reduction to encompass cardiovascular, hepatic, inflammatory, and reproductive health. Leptin now emerges as a foundational biological signal whose clinical relevance can be realized through precise, multimodal, and mechanism-guided methods for obesity and obesity-related disorders. In this light, the emergence of obesity care lies in unmasking the therapeutic capacity of this regulatory signal to reclaim systemic health.

## Declaration

### Acknowledgments

This work was funded by the Ministry of Science, Technological Development, and Innovation of the Republic of Serbia (Contract No# 451-03-33/2026-03/200017).

### Conflict of interest

The authors declare that they have no conflict of interest.

### Author contribution

M.O., S.K. and S.Z. - writing - original draft, E.R.I. - writing, conceptualization and supervision.

### Artificial Intelligence (AI) - assisted technology

The authors declare that generative AI was not used in the creation of this manuscript.
